# 1D and 2D-HSQC NMR: Two Methods to Distinguish and Characterize Heparin From Different Animal and Tissue Sources

**DOI:** 10.3389/fmed.2019.00142

**Published:** 2019-06-27

**Authors:** Lucio Mauri, Maria Marinozzi, Nisarga Phatak, Michael Karfunkle, Kalib St. Ange, Marco Guerrini, David A. Keire, Robert J. Linhardt

**Affiliations:** ^1^NMR Center, Istituto di Ricerche Chimiche e Biochimiche “G. Ronzoni,” Milan, Italy; ^2^Division of Pharmaceutical Analysis, Office of Testing and Research, Center for Drug Evaluation and Research, U.S. Food and Drug Administration, St. Louis, MO, United States; ^3^Department of Chemistry and Chemical Biology, Center for Biotechnology and Interdisciplinary Studies, Rensselaer Polytechnic Institute, Troy, NY, United States

**Keywords:** heparin, NMR, HSQC, pharmacopeia, animal origin

## Abstract

The US Food and Drug Administration has encouraged the reintroduction of bovine heparin drug product to the US market to mitigate the risks of heparin shortages and potential adulteration or contamination of the primary source which is porcine heparin. Here, a 1D-NMR method was applied to compare heparin sodium of bovine intestinal origin with that of bovine lung, porcine, or ovine intestinal origin. The results showed that a simple 1D test using NMR signal intensity ratios among diagnostic signals of the proton spectra uniquely identified the origin of heparin and concomitantly could be used to assure the correct sample labeling. However, a limitation of the use of only mono-dimensional spectra is that these spectra may not provide sufficiently detailed information on the composition of heparin batches to adequately determine the quality of this complex product. As an alternative, a higher resolution quantitative 2D-HSQC method was used to calculate the percentage of mono- and disaccharides, distinguish the origin of heparin and, simultaneously, assess the heparin composition. The 2D-HSQC method is proposed to provide sufficient information to evaluate the quality of industrial production process used to make the drug substance. Together, the 1D and 2D data produced by these measurements can be used to assure the identity and purity of this widely used drug.

## Introduction

Heparin, one of the world's most widely-sold polysaccharide drugs and included in the World Health Organization's “Essential Drugs List” (1), is used for the treatment and prevention of thrombosis and is the anticoagulant of choice during cardio-pulmonary bypass or for hemodialysis. Until the 50's, bovine lung was the main source material for the large-scale heparin production, after which bovine lung tissue was largely replaced by porcine mucosa (2). Subsequently, in the late 1980s there was a spike in the number of cases of bovine spongiform encephalopathy (BSE) in the United Kingdom. As a result of concerns with contamination of the heparin supply, bovine heparin was voluntarily withdrawn from the market, and currently porcine mucosa is the only source material for all heparin products approved in US and Europe (3).

Following the “heparin crisis” of 2007–2008, when at least 81 deaths and hundreds of severe anaphylactoid reactions were linked to the presence of over-sulfated chondroitin sulfate (OSCS) in some heparin batches, FDA and EMA initiated a rapid revision of the existing pharmacopeia monographs (4, 5). In addition to including tests regarding the potency and more restrictive limits for impurities, proton nuclear magnetic resonance (1H-NMR) was introduced as an identity test. The NMR test allowed one to define the similarity of the test sample relative to a porcine heparin reference standard, as well as to examine if non-heparin signals could be possibly associated with impurities or contaminants (6).

Because China is responsible for roughly half of the world's pig production, about 60% of the heparin supply comes from this country. In recent years the US FDA has expressed significant interest in considering reintroduction of bovine heparin drug product to the US market to mitigate the risk of supply chain issues due to the lack of source material and to diversify the source of heparin drugs globally (7). The FDA, assisted by experts coming from non-profit organizations, industries and academic institutions, is investigating the structural differences between porcine, and bovine heparins and their possible clinical implications. Previous studies extensively demonstrated structural differences between heparin from different animal and organ sources. Particularly, porcine mucosa heparin (PMH) is characterized by higher 6-*O*-sulfation compared to bovine mucosa heparin (BMH) (80% in PMH vs. 55% in BMH comparing the total amount of GlcNx,6S or 68% in PMH vs. 48% in BMH comparing the amount of GlcNS, 6S linked to IdoA2S) (8, 9) and different structures and distribution of antithrombin binding sequences (10). Despite the same animal source, bovine lung heparin (BLH) differs from BMH with higher 2-*O-*, 6-*O*-, and *N*-sulfation and lower content of GlcA and GlcNAc residues (11, 12). By contrast, Ovine mucosa heparin (OMH) is more similar to PMH, with the exception of slightly higher levels of sulfation in position 2 and 6 of glucosamine and in position 2 of iduronic acid and lower N-acetylation (13, 14).

In 2017 bovine mucosal heparin was readmitted to the market in Brazil, requiring the drafting of a new specific monograph. The NMR identification test of this new monograph includes an acceptance criterion for BMH corresponding to the ratio among the integral values of anomeric signals of glucosamine 6-*O*-sulfated and 6-*O*-desulfated which is not satisfied by PMH spectra (15). In the present study an alternative method based on the measurements of the ratio between the intensity of diagnostic signals of the ^1^H-NMR spectrum was proposed. The method proposed here can distinguish BMH, PMH, OMH, and BLH samples from one another, while the ANVISA approach is specific for BMH vs. PMH.

Recently, quantitative 2D-heteronuclear single quantum coherence (HSQC) NMR spectroscopy was established as viable method to determine the mono and disaccharide composition of heparin and LMWH (16, 17). The study of the factors influencing the limit of detection, linearity, accuracy, and precision demonstrated the robustness of the method in assessing the quality of heparin (18). This method, calculating the percentage of monosaccharide and disaccharide building blocks, can distinguish the heparin source as well as identify possible structural modification of the heparin chains induced by the purification process, often undetectable in one-dimensional NMR data. The HSQC method, applied to libraries of BMH, PMH, OMH, and BLH heparin batches coming from different suppliers provided a basis set of characteristics for each heparin source and of their structural variability.

## Materials and Methods

### Reagents and Starting Material

Deuterium oxide 99.9%, sodium dihydrogenphosphate hydrate (NaH_2_PO_4_·H_2_O), disodiumhydrogen phosphate dihydrate (Na_2_HPO_4_·H_2_O_2_), and 3-(trimethylsilyl)propionic-2,2,3,3-d4 acid sodium salt (TSP) were purchased from Sigma-Aldrich (Milan, Italy). Deuterated EDTA d-16 98% was obtained from Product Cambridge Isotope Laboratories, Inc.

Phosphate buffer solution was prepared as follows: 49.7 mg of sodium dihydrogenphosphate hydrate (0.36 mmol), 202.9 mg of disodium hydrogen phosphate dihydrate (1.14 mmol), and 0.92 mg deuterated EDTA d-16 (0.003 mmol) were dissolved in 10 mL of water. The pH was checked at 7.1. The solution was distributed into 5 mL aliquots and then lyophilized. Each aliquot was dissolved in about 1 mL of D2O and lyophilized again. Finally, the buffer was dissolved in 5 mL of deuterium oxide with 0.002% TSP (12 mM).

### Heparin Samples

The heparin samples utilized in this study were obtained from 13 different producers. The samples included 39 porcine mucosa heparins, 39 bovine mucosa heparins, 6 ovine mucosa, and 7 from beef lung heparins. 20 BMH, 2 PMH, and 1 BLH were provided by USP, the remaining samples from Ronzoni Institute.

All the samples were characterized by ^1^H and HSQC-NMR analysis. Table 1 summarizes the ^1^H-NMR spectra recorded for this study.

**Table 1:** Number of 1H-NMR spectra per heparin origin and spectrometer frequency.

	**BMH**	**PMH**	**OMH**	**BLH**
^1^H at 500 MHz	39	39	6	7
^1^H at 600 MHz	20	19	6	7

### Samples Preparation

About 35 mg of heparin sample were dissolved in a 0.6 ml of phosphate buffer solution and transferred on a 5 mm NMR tube.

### ^1^H-NMR Method

^1^H-NMR spectra were measured on a Bruker AVANCE III 600 MHz spectrometer or on a Bruker AVANCE III HD 500 MHz spectrometer (Karlsruhe, Germany), equipped with 5 mm TCI cryogenic probes. The experiments were recorded at 298 K by using the Bruker library zg pulse sequence and the following parameters: number of scans 16, dummy scans 4, relaxation delay 12 s, spectral width 16 ppm, transmitter offset 4.7 ppm. After exponential multiplication (line broadening of 0.3 Hz), the spectra were Fourier transformed, phased, baseline corrected, and calibrated on the TSP signal.

The spectrum can be accepted only if the following test is satisfied: the width half height of TSP is ≤ 1.4 Hz.

The intensity of the following signals was measured:

- Peak 1 at 3.87 ppm that corresponds to the H5/H6 of the *N*-sulfated, 6-*O*-desulfated glucosamine (GlcNS, 6OH).
- Peak 2, the highest intensity signal between 3.33 and 3.20 ppm, that corresponds to the H2 of *N*-sulfated glucosamine (GlcNS,6X).
- Peak 3 at 2.05 ppm that correspond to the methyl signal of *N*-acetylated glucosamine (COCH_3_).

The ratio r(1:2) between peaks 1 and 2, and the ratio r(3:2) between peaks 2 and 3 were calculated and used to distinguish the different heparin sources.

### HSQC-NMR Method

The 2D-^1^H,^13^C-HSQC spectra were measured on Bruker AVANCE III 600 MHz spectrometer equipped with a 5 mm TCI cryoprobe, using the Bruker hsqcetgpsisp2.2 pulse sequence. The spectra were recorded at 298 K using the following acquisition parameters: number of scans 12, dummy scan 16, relaxation delay 2.5 s, spectral width 8 ppm (F2), and 80 ppm (F1), transmitter offset 4.7 ppm (F2) and 80 ppm (F1), ^1^J_C−H_ = 150 Hz. 1,024 points were recorded for each of 240 increments (NUS of 75% of 320 increments). The FIDs were processed as follows: spectrum size 4096 (F2) and 1,024 (F1) (zero-filling in F2 and linear prediction in F1), squared cosine window multiplication in both dimensions and Fourier transform. The spectra were integrated using Topspin software version 3.5 (Bruker BioSpin, Rheinstetten, Germany) and the heparin composition was computed from the integral values as previously described (18).

### Statistical Methods

Prediction intervals were computed assuming normal distributions (Kolmogorov-Smirnov tests gave *p* > 0.3) of the data, using the formula:

$$\begin{array}{l} \left(\bar{x}-\tau_{0.995,n-1}\cdot sd\cdot \sqrt{1+1/n},\;\bar{x}+\tau_{0.995,n-1}\cdot sd\cdot \sqrt{1+1/n}\right), \end{array}$$

where *x* and *sd* are the mean and the standard deviation of the *n* observations, and τ_0.995,*n*−1_ is the 0.995th quantile of the Student's *t*-distribution with *n* − 1 degrees of freedom.

Boxplot were produced as follows: the lower and upper limits of the box correspond to the 25th and the 75th percentiles. Called *IQR* the distance between these two limits, the ends of the boxplot vertical segments correspond to the lowest datum still within 1.5 IQR of the 25th percentile, and the highest datum still within 1.5 IQR of the 75th percentile. Data outside of the vertical segments were plotted as dots.

## Results

**Figure 1:**
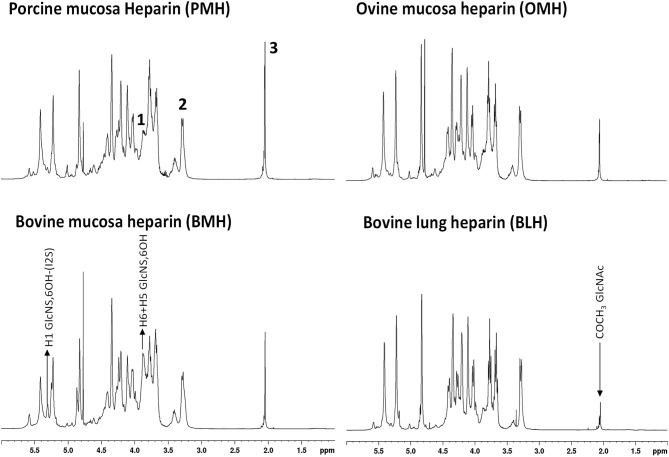
600 MHz proton spectra of heparin samples extracted from different species/organ. Signals 1, 2, and 3, corresponding to H5 + H6 of 6-*O*-desulfated glucosamine, H2 of *N*-sulfated glucosamine, and acetyl group of *N*-acetylated glucosamine, respectively, were used as diagnostic peaks for the determination of the origin.

Proton NMR spectra of heparin show slightly different profiles according the animal/organ origin (Figure 1). Of the types tested here, proton NMR spectra of BMH have the most observable differences compared to other heparins. Particularly, the heparin signals at 5.31 and 3.87 ppm, corresponding to the H1 and H5/H6 of the 6-*O*-desulfated glucosamine, respectively, had greater intensities in BMH spectra compared to those observed in the spectra of other heparin sources. This observation was consistent with the observation of lower 6-*O*-sulfation levels in bovine mucosa heparin described in previous studies (8, 19). The spectrum of bovine lung heparin (BLH) showed structural peculiarities compared other spectra, particularly evident in the lower intensity of the acetyl peak at 2.05 ppm. By contrast, proton spectra of PMH and OMH were more similar, even though a reduction of the acetyl peak intensity can be observed in the spectra of OMH compared to PMH data.

**Figure 2:**
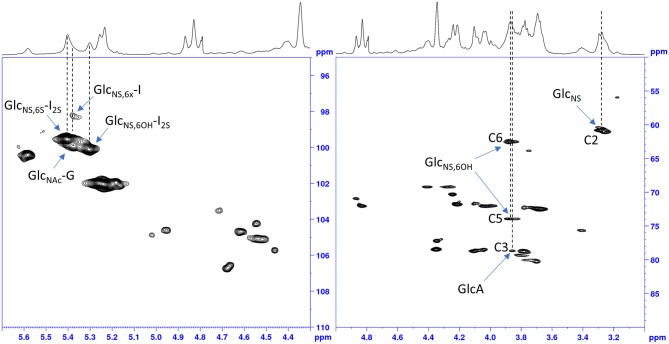
HSQC spectrum of a bovine heparin. Expansions of the anomeric signals region **(left)** and of ring signals **(right)**.

The idea to integrate diagnostic signals of carbon and proton NMR spectra to distinguish bovine and porcine heparin was first proposed by Casu et al. (8) and then by Tovar et al. (20) and Fu et al. (14). The identification test for bovine mucosa heparin, currently used by the drug regulatory agency of Brazil (15), uses the ratio between the integral values of proton anomeric signals belonging to glucosamine 6-*O*-sulfated and 6-*O*-desulfated residues, with chemical shifts at 5.40 and 5.31 ppm, respectively. However, because the anomeric proton signal of GlcNS,6S used by the ANVISA method, at least at magnetic field lower than 600 MHz, partially overlaps with those of *N*-sulfated glucosamine linked to iduronic acid (GlcNS-I) and *N*-acetyl glucosamine linked to glucuronic acid (GlcNAc-G) (Figure 2), we decided to use different signals which had less interference from other monosaccharides. As reference signals we chose the H2 of *N*-sulfated glucosamine (GlcNS) and the acetyl signal of *N*-acetylated glucosamine (GlcNAc) which have chemical shift values of 3.28 and 2.04 ppm, respectively, and are well separated from the other signals (annotated peaks 2 and 3 of Figure 1). For a marker for the 6-*O*-desulfated glucosamine population, typical of BMH, the H5 and H6 protons signals (3 protons) were used and were overlapped in the same peak at 3.87 ppm (peak 1 of Figure 1). Although the 3.87 ppm signal was also partially superimposed with the signal from H3 of the glucuronic acid (GlcA) (21), the sensitivity was better than that obtained from the use of the anomeric signal, which was due to just one proton (Figure 2).

**Figure 3:**
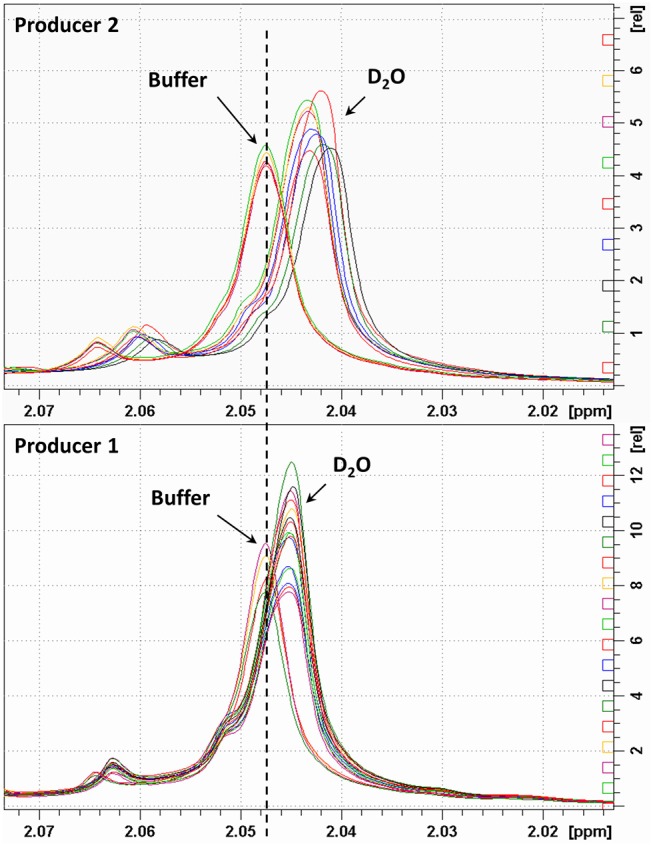
Acetyl signal of heparin 1H-NMR spectra registered in D_2_O or buffer. Top and bottom panels show samples of two different manufacturers.

The integral values of partially overlapped heparin signals can vary as a function of the magnetic field used, spectral resolution or by the natural structural variability of heparin (22). Moreover, the ANVISA method was designed to distinguish BMH from PMH, while the method discussed here aims to differentiate PMH, BMH, OMH, and BLH. For this reason, we evaluated the signal intensities instead of the integral values. Due to the polyelectrolyte characteristic of heparin, slight differences in the concentration, pH or ionic strength of the heparin solutions can induce small changes in chemical shift values. Figure 3 shows the acetyl signal of the proton spectra of several PMH samples from two different suppliers, measured in deuterium oxide or in phosphate buffer. The addition of buffer removes the small shifts observed in deuterium oxide, making the spectra more reproducible, and comparable by minimizing pH and ionic strength sources of variation. Moreover, to avoid variation of signal intensities due to bad spectral resolution from poor field homogeneity, a requirement of a minimum full width at half-height linewidth of the signal of deuterated trimethylsilylpropionic acid sodium salt (TSP), used as chemical shift reference, was introduced (see the Experimental Section).

### 1H-NMR Spectra

**Figure 4:**
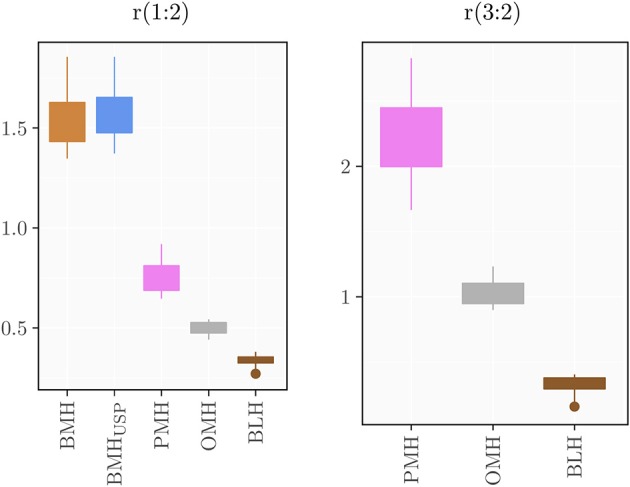
Boxplots of the computed ratios r(1:2) and r(3:2) for the different heparin sources.

**Figure 5:**
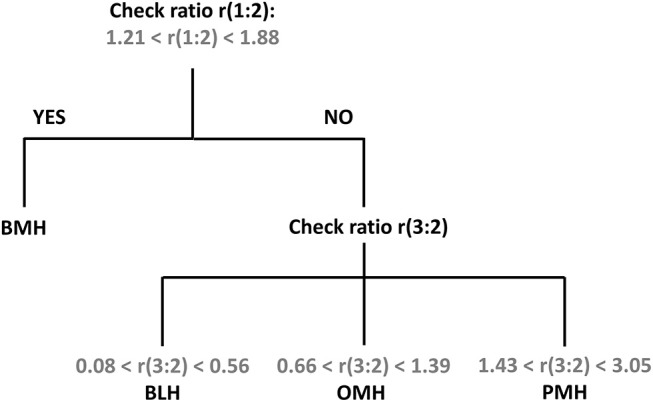
Proposed scheme for the identification of heparin source from the signal ratios r(1:2) and r(3:2).

Using the standardized conditions described in the experimental section, 39 BMH, 39 PMH, 6 OMH, and 7 BLH samples were analyzed with the proposed ^1^H-NMR method at 500 and/or 600 MHz. The intensities of peaks 1 (3.87 ppm), 2 (3.28 ppm), and 3 (2.05 ppm) were measured and the ratios 1:2 and 3:2 were calculated (Supplementary Tables 1–6). Results, shown in Figure 4, indicate that a ratio of 1:2 clearly distinguishes BMH from the other heparin sources, whereas a ratio of 3:2 differentiates PMH, OMH, and BLH. Accordingly, the scheme presented in Figure 5 can be used to identify heparin types from each group from these ratios: first check the 1:2 ratio is between 1.21 and 1.88, to classify the sample as BMH, otherwise check the 3:2 ratio to decide among PMH, OMH, BLH, or an unknown source. The limits of Figure 5 correspond to prediction intervals with 99% probability (see Experimental section). The PMH and OMH interval separation was the smallest, confirming the similarity of heparins from these two sources. The results were only marginally affected by the magnetic field strength (500 or 600 MHz) so the scheme can be adopted independently of the instrument used.

A repeatability study was performed using a PMH heparin batch to determine the precision of the method. Six NMR tubes of the same sample were prepared, and proton spectra were recorded on each sample using 16 or 8 scans, respectively. Results summarized in the Supplementary Table 7 show that the CV% of both ratios was ≤4% regardless the number of scans.

**Figure 6:**
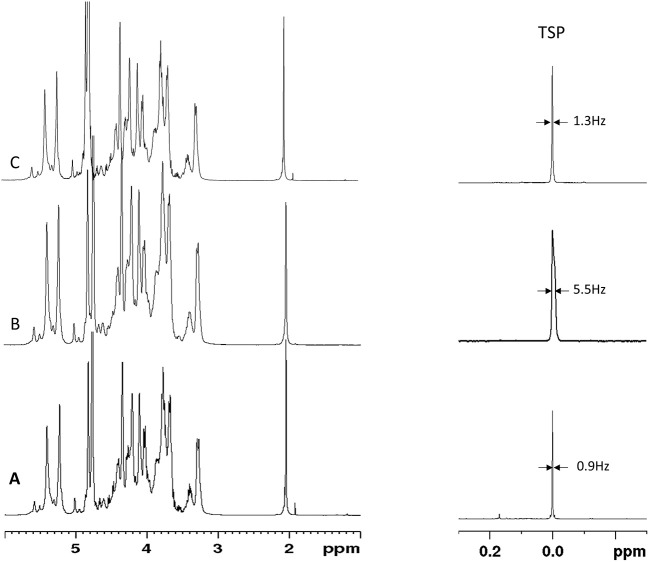
Proton spectra of PMH sample provided by USP measured by Laboratory 1 **(A)** and Laboratory 2 **(B,C)**. TSP signal and the corresponding width at half height is reported.

A series of 20 BMH, 1 PMH, and 1 BLH provided by USP were analyzed by ^1^H-NMR by two independent laboratories. Results obtained on BMH show a good comparability between the ranges and values obtained by the two laboratories (Table 2 and Supplementary Table 8). This was confirmed by a paired *t*-test on the r(1:2) value of the BMH samples which gave a *p*-value of 0.45. Both laboratories identified test samples (BLH and PMH) as non-compatible with BMH [r(1:2) ≤ 1]. However, while the BLH sample was correctly identified by both laboratories, laboratory 2 identified the PMH sample as OMH, since r(3:2) value (1.37) was within the range of mucosa ovine heparin. A more detailed analysis of the spectra reveals a lower spectral resolution of the PMH spectrum measured by laboratory 2, confirmed by the different width at half height of TSP between the two spectra (about 1 Hz and 5 Hz, for laboratory 1 and 2, respectively) (Figures 6A,B). The r(3:2) ratio value obtained from the spectrum measured by laboratory 2 on the same sample, comply with the resolution requirement (width at half height of TSP of 1.4 Hz), was 2.15, therefore compatible with PMH sample (Figures 5, 6C). This confirms that the evaluation of the spectrum resolution using the width half height of the TSP signal is crucial for a correct identification of the heparin sources with the proposed method.

**Table 2:** Range of signal intensity ratios calculated on 20 BMH samples by two laboratories.

		**Range**	**BMH**	**PMH sample**	**BLH sample**
		**min**	**max**		
r(1:2)	Lab 1	**1.35**	**1.86**	0.74	0.38
	Lab 2	**1.40**	**1.85**	0.72	0.35
r(3:2)	Lab 1			2.30	0.40
	Lab 2			1.37*/2.15	0.34

One PMH and one BLH sample were used as test samples. *value measured on PMH by laboratory 2 from spectrum which does not comply with resolution requirement. r_(1:2) of the BMH samples measured by two different laboratories are shown in bold.

### HSQC Analysis

Quantitative HSQC was recently applied to heparin and low molecular weight heparins (LMWHs) for calculating the percentage of monosaccharides and disaccharides by normalizing volumes with reference to the sum of volumes of signals corresponding to each monosaccharide type (glucosamines or uronic acids) and the same carbon proton pair type (17, 18). This validated analytical procedure can be used to assess the quality of industrial production in terms of presence of contaminants or chemical modifications, as well as to compare heparin of different manufacturers or to differentiate heparin of different sources (10).

**Figure 7:**
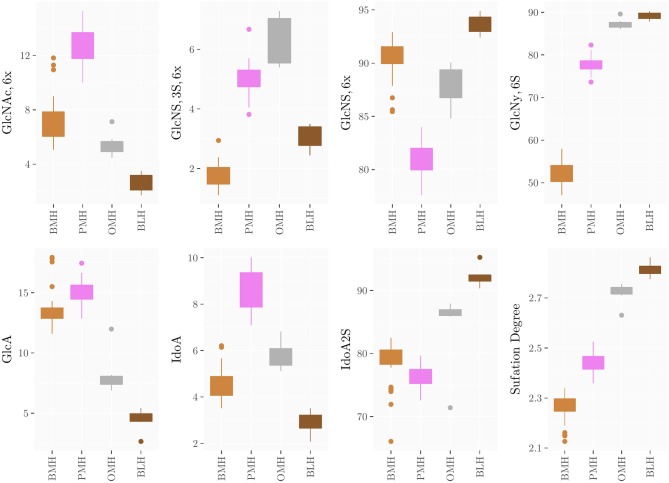
Boxplots of the monosaccharides composition of heparins from the different sources obtained by HSQC analysis.

The method was applied to all groups of heparins and results are reported in Supplementary Materials (Supplementary Tables 9–21) and summarized in the box plots of Figure 7. Notably, the monosaccharide composition of both glucosamine and uronic acid shows some variability within the same heparin source that is attributed to the natural variability of the starting material and to the different process conditions used by manufacturers to purify heparin from the crudes. However, the process related variability is much lower than the differences found among the heparins of different origin (Figure 7). The strong reduction of 6-*O*-sulfation of BMH compared to the other heparin sources (GlcNy,6S < 60%), allows to easily differentiate BMH from other heparin types, similar to previously described carbon spectrum integration methods (8).

Importantly, differences which were not observable in the 1D proton spectra were clearly detected by the HSQC method. For instance, OMH, PMH, and BLH differ not only in the degree of acetylation of glucosamine residues (PMH>OMH>BLH) but also in the glucuronic acid content and non-sulfated iduronic acid content (PMH>OMH>BLH) (Figure 7). The range of values of the corresponding residues can then be used to distinguish each heparin source.

A further level of characterization involved the quantification of disaccharide sequences and minor features, such as the heparin biological activity related glucuronic acid linked to 3-*O*-sulfated glucosamine (G-A^*^) disaccharide that is a marker of the antithrombin-binding (ATb) pentasaccharide (23).

Because the G-A^*^ sequence was identified exclusively in the active pentasaccharide sequence, G-A^*^ can be considered the marker of the heparin ATb site. The amount of G-A^*^ has a certain variability within each heparin source (Supplementary Tables 11, 14, 17, 20; Supplementary Figures 1, 2), which together with the molecular weight, may explain potency variability observable in commercial products. In contrast, the G-A^*^ content varies considerably among heparin obtained by different sources. In particular, the highest amount of G-A^*^ was observed in the PMH samples (from 1.9 to 3.4%), while in the other species G-A^*^ was always lower than 1.0 %, with the exception of one ovine sample for which the G-A^*^ value reached 2.8%. Moreover, in several BMH samples G-A^*^ residue was not detectable (below LOD), possibly due to the presence of alternative AT-binding sequences (10).

In addition, the presence of 2,3-epoxy gluonic acid (epoxide) and galacturonic acid were observed and are known to be formed during the alkali treatment of heparin that occurs during the heparin purification process (24). Even if epoxide and galacturonic acid residues were not detected in many batches (Supplementary Tables 11, 14, 17, 20; Supplementary Figures 1, 2), some samples contained levels of these unnatural residues which were not observable in proton NMR spectra. Notably, 15 of 39 BMH samples contain galacturonic acid ranging from 0.9 to 9.8% and 4 of 39 BMH samples contain epoxide (1.3to 4.8%). These large amounts of chemically modified structures can affect not only the activity of heparin but possibly also the pharmacokinetic and other biological properties and therefore the control of epoxide and galacturonic acid process related impurities levels in the heparin API manufacturing process would be recommended.

## Conclusion

In response to the FDA interest in considering reintroduction of bovine heparin drug product to the US market, there is a need to develop tests for the identity of the heparin animal and tissue origin. The method developed here, is an alternative to that proposed by ANVISA, that is able not only to distinguish BMH from PMH, but also to confirm that the sample was not manufactured from other heparin sources such as bovine lung or ovine mucosa. The method, based on measurement of a proton NMR spectrum, is not much different from that present in both US and EU pharmacopeias for the identification of PMH heparin. A method utilizing the intensity ratios among selected signals was indeed already used in several pharmacopeia monographs to detect the presence of possible unknown contaminant (7). Here, a further proposed modification allows more different heparin types from potentially commercially viable animal sources to be distinguished, making clear the source of the product.

Nevertheless, overlapping signals on the proton NMR spectra prevent signals of minor structural signatures to be detected. For example, the presence of epoxidated or galacturonic acid residues cannot be easily observed in 1D proton spectra, while their signals are well resolved in the 2D HSQC spectra. The possibility to use the HSQC spectra, not only to assign 1D superimposed proton signals but also to quantify the relative abundance of each residue of glycosaminoglycans, was recently proposed by different groups (17, 18). These groups showed that a single HSQC analysis was able to provide the mono and disaccharide composition of heparin, including detection, and quantification of process related residues (i.e., epoxidation, galacturonic acid, or residues generated by oxidative treatment) (24–26) establish the species and organ source of heparin and detect the presence of possible contaminants. The presence and the identification of the contaminant (OSCS) during the heparin crisis was indeed confirmed by HSQC analysis (4).

Even if 2D HSQC would be preferable in terms of amount of information obtained, the need for instruments equipped with high sensitivity probes (for example, cryogenically cooled probes) and for operators skilled in NMR, will make the application of the HSQC method difficult for many analytical laboratories. Although lower resolution and containing less information than 2D data sets, 1D proton-NMR data can be used to distinguish heparin types in a facile manner and is very similar to a method that is already required by several pharmacopeias and more widely applicable to an analytical laboratory. However, the increasing sensitivity and accuracy of NMR spectrometers and the availability of software which allows the complete automatization of the spectra processing and analysis have made the HSQC methodology a viable information value added alternative for many laboratories and may become more widely accessible in the future.

## Ethics Statement

The study described in the manuscript does not involve human subjects.

## Author Contributions

LM, MG, DK, and RL contributed to planning and writing the paper. LM, MM, KS, MK and NP performed the NMR analyses. LM and MK performed the statistical analysis. All authors contributed to revision of the manuscript and read and approved the submitted version.

### Conflict of Interest Statement

The authors declare that the research was conducted in the absence of any commercial or financial relationships that could be construed as a potential conflict of interest.
