# Supplementary material for: 1D and 2D-HSQC NMR: Two Methods to Distinguish and Characterize Heparin From Different Animal and Tissue Sources
Source: Front Med (Lausanne). 2019 Jun 27;6:142. doi: 10.3389/fmed.2019.00142 (PMC6610300; doi:10.3389/fmed.2019.00142)
Supplement: Supplementary file 1 [file Presentation_1.pdf]

*Supplementary material*

# 1D and 2D-HSQC NMR: two methods to distinguish and characterize heparin from different animal and tissue sources.

Lucio Mauri[1], Maria Marinozzi[1], Nisarga Phatak[2], Michael Karfunkle[2], Kalib StAnge[3], Marco Guerrini[1]*, David A. Keire[2]*, Robert J. Linhardt[3]*

[1] Istituto di Ricerche Chimiche e Biochimiche "G. Ronzoni", Milan, Italy.
[2] Division of Pharmaceutical Analysis, Office of Testing and Research, Center for Drug Evaluation and Research, U.S. Food and Drug Administration, St. Louis, MO, USA
[3] Department of Chemistry and Chemical Biology, Center for Biotechnology and Interdisciplinary Studies, Rensselaer Polytechnic Institute, Troy, New York.

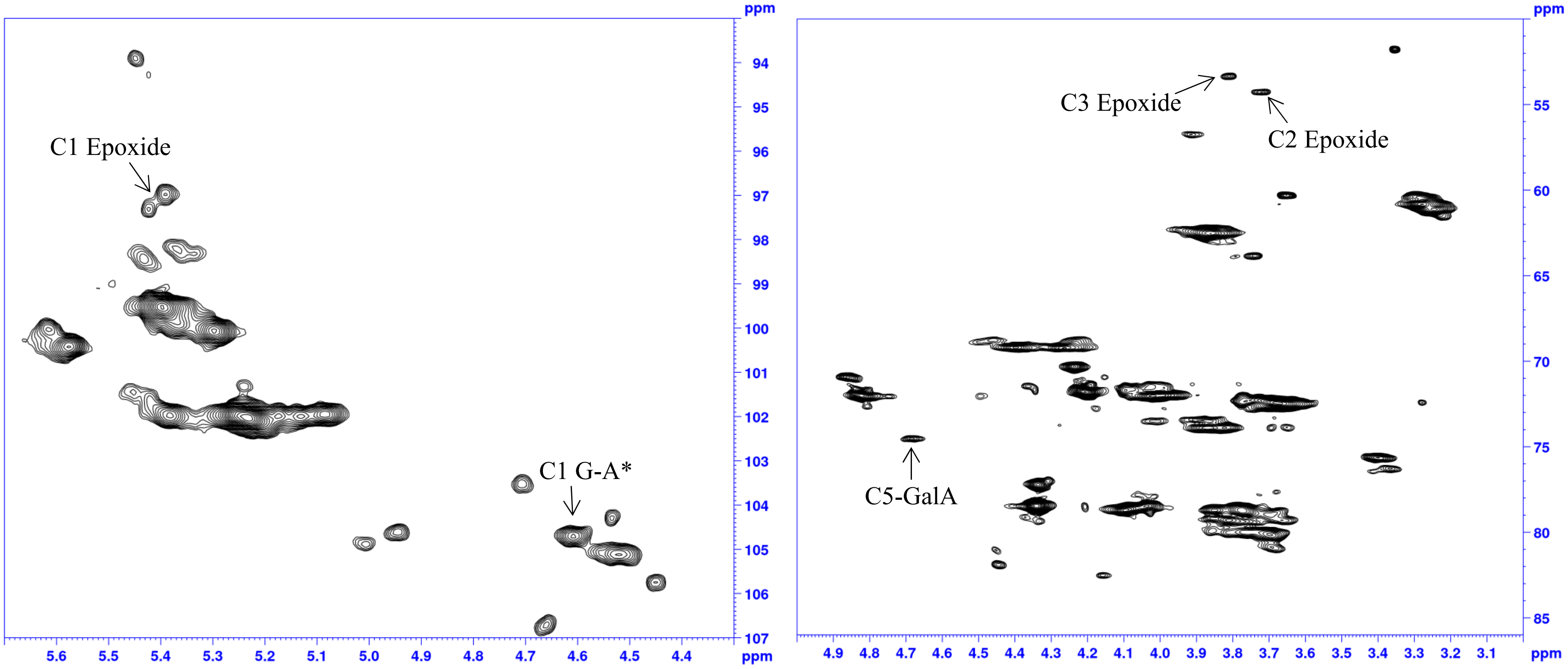


**Figure S1** – HSQC spectrum of beef mucosa heparin containing epoxide and galacturonic acid residues. Expansions of the anomeric signals region (left) and of the ring signals (right).

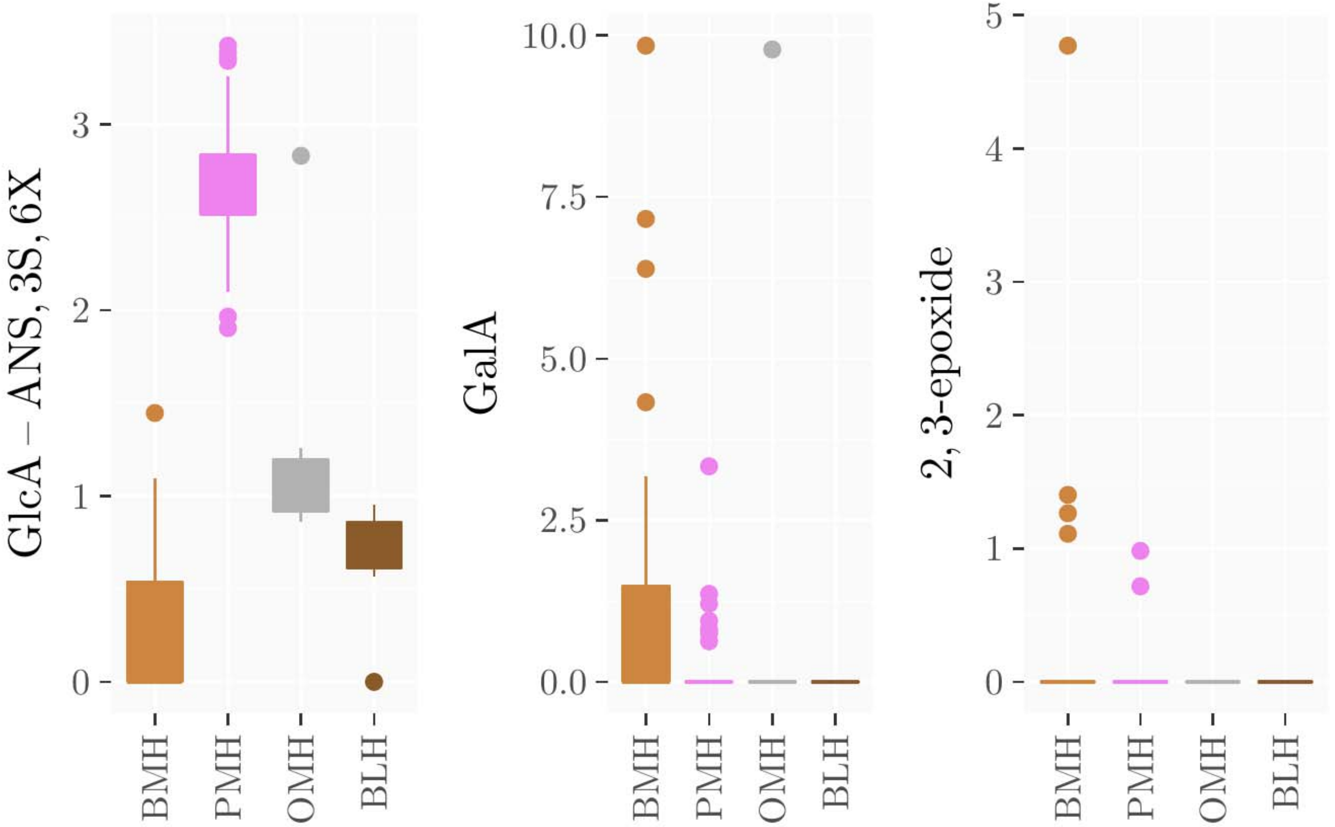


**Figure S2-** Boxplot of the HSQC content of G-($A_{NS,3S,6X}$), galacturonic acid and epoxide residues**.**

**Table S1-** Signal intensity ratio calculated on BMH spectra recorded at 500MHz

| Sample | r (1:2) | r (3:2) |
|---|---|---|
| BMH-1 | 1.64 | 1.79 |
| BMH-2 | 1.53 | 1.49 |
| BMH-3 | 1.55 | 1.44 |
| BMH-4 | 1.55 | 1.47 |
| BMH-5 | 1.53 | 1.45 |
| E1 | 1.53 | 1.98 |
| F1 | 1.51 | 1.79 |
| G1 | 1.48 | 1.86 |
| H1 | 1.4 | 1.74 |
| E2 | 1.73 | 2.56 |
| F2 | 1.78 | 2.83 |
| G2 | 1.86 | 2.73 |
| K1 | 1.6 | 1.59 |
| L1 | 1.48 | 1.44 |
| M1 | 1.43 | 1.26 |
| N1 | 1.38 | 1.29 |
| O1 | 1.41 | 1.36 |
| P1 | 1.48 | 1.33 |
| Q1 | 1.52 | 1.55 |
| R1 | 1.53 | 1.54 |
| S1 | 1.6 | 1.48 |
| O2 | 1.65 | 1.48 |
| P2 | 1.64 | 1.49 |
| Q2 | 1.62 | 1.44 |
| R2 | 1.62 | 1.44 |
| BMH-6 | 1.42 | 1.7 |
| BMH-7 | 1.53 | 1.97 |
| BMH-8 | 1.49 | 1.8 |
| BMH-9 | 1.6 | 1.9 |
| BMH-10 | 1.64 | 2.06 |
| BMH-11 | 1.69 | 2.01 |
| BMH-12 | 1.43 | 1.56 |
| BMH-13 | 1.47 | 1.71 |
| BMH-14 | 1.42 | 1.67 |
| BMH-15 | 1.39 | 1.71 |
| BMH-16 | 1.35 | 1.7 |
| BMH-17 | 1.44 | 1.76 |
| BMH-18 | 1.35 | 1.66 |
| BMH-19 | 1.41 | 1.63 |
| MIN | 1.4 | 1.3 |
| MAX | 1.8 | 2.9 |

**Table S2-** Signal intensity ratio calculated on BMH spectra recorded at 600MHz

| Sample | r (1:2) | r (3:2) |
|---|---|---|
| E1 | 1.54 | 2.06 |
| F1 | 1.51 | 1.81 |
| G1 | 1.48 | 1.85 |
| H1 | 1.4 | 1.76 |
| E2 | 1.73 | 2.92 |
| F2 | 1.77 | 2.9 |
| G2 | 1.84 | 2.81 |
| K1 | 1.59 | 1.65 |
| L1 | 1.48 | 1.37 |
| M1 | 1.41 | 1.3 |
| N1 | 1.37 | 1.28 |
| O1 | 1.39 | 1.37 |
| P1 | 1.47 | 1.38 |
| Q1 | 1.51 | 1.52 |
| R1 | 1.52 | 1.58 |
| S1 | 1.59 | 1.5 |
| O2 | 1.68 | 1.6 |
| P2 | 1.68 | 1.6 |
| Q2 | 1.66 | 1.59 |
| E1 | 1.68 | 1.55 |
| MIN | 1.37 | 1.28 |
| MAX | 1.84 | 2.92 |

**Table S3-** Signal intensity ratio calculated on PMH spectra recorded at 500MHz

| Sample | r (1:2) | r (3:2) |
|---|---|---|
| PMH-1 | 0.66 | 2 |
| PMH-2 | 0.71 | 2.23 |
| PMH-3 | 0.7 | 2.26 |
| PMH-4 | 0.7 | 2.15 |
| PMH-5 | 0.77 | 2.26 |
| PMH-6 | 0.89 | 2.69 |
| PMH-7 | 0.77 | 2.43 |
| PMH-8 | 0.71 | 2.14 |
| PMH-9 | 0.8 | 2.57 |
| PMH-10 | 0.81 | 2.6 |
| PMH-11 | 0.89 | 2.36 |
| PMH-12 | 0.72 | 2 |
| PMH-13 | 0.85 | 2.7 |
| PMH-14 | 0.82 | 2.63 |
| PMH-15 | 0.86 | 2.69 |
| PMH-16 | 0.81 | 2.6 |
| PMH-17 | 0.86 | 2.7 |
| PMH-18 | 0.92 | 2.83 |
| PMH-19 | 0.68 | 1.97 |
| PMH-20 | 0.74 | 2.4 |
| PMH-21 | 0.71 | 1.81 |
| PMH-22 | 0.67 | 1.66 |
| PMH-23 | 0.83 | 2.54 |
| PMH-24 | 0.75 | 2.27 |
| PMH-25 | 0.74 | 2.3 |
| PMH-26 | 0.7 | 1.98 |
| PMH-27 | 0.71 | 2.02 |
| PMH-28 | 0.71 | 1.79 |
| PMH-29 | 0.79 | 2.55 |
| PMH-30 | 0.66 | 1.75 |
| PMH-31 | 0.68 | 1.84 |
| PMH-32 | 0.87 | 2.79 |
| PMH-33 | 0.82 | 2.5 |
| PMH-34 | 0.68 | 2 |
| PMH-35 | 0.68 | 1.73 |
| PMH-36 | 0.72 | 1.85 |
| PMH-37 | 0.72 | 1.86 |
| PMH-38 | 0.71 | 1.95 |
| PMH-39 | 0.75 | 2.23 |
| MIN | 0.66 | 1.66 |
| MAX | 0.92 | 2.83 |

**Table S4-** Signal intensity ratio calculated on PMH spectra recorded at 600MHz

| Sample | r (1:2) | r (3:2) |
|---|---|---|
| PMH-1 | 0.65 | 1.97 |
| PMH-2 | 0.67 | 2.12 |
| PMH-3 | 0.65 | 2.13 |
| PMH-4 | 0.65 | 2.08 |
| PMH-5 | 0.73 | 2.18 |
| PMH-6 | 0.83 | 2.57 |
| PMH-7 | 0.76 | 2.12 |
| PMH-8 | 0.66 | 1.94 |
| PMH-9 | 0.74 | 2.34 |
| PMH-10 | 0.76 | 2.37 |
| PMH-11 | 0.85 | 2.31 |
| PMH-12 | 0.67 | 1.86 |
| PMH-13 | 0.78 | 2.44 |
| PMH-14 | 0.77 | 2.41 |
| PMH-15 | 0.81 | 2.45 |
| PMH-16 | 0.76 | 2.37 |
| PMH-20 | 0.67 | 2.22 |
| PMH-25 | 0.67 | 2.12 |
| PMH-39 | 0.7 | 2.09 |
| MIN | 0.65 | 1.86 |
| MAX | 0.85 | 2.57 |

**Table S5-** Signal intensity ratio calculated on OMH spectra recorded at 500MHz (a) and 600MHz (b)

a)

| Sample | r (1:2) | r (3:2) |
|---|---|---|
| OMH-1 | 0.54 | 0.96 |
| OMH-2 | 0.48 | 1.01 |
| OMH-3 | 0.52 | 1.02 |
| OMH-4 | 0.48 | 0.98 |
| OMH-5 | 0.54 | 1.23 |
| OMH-6 | 0.52 | 1.21 |
| MIN | 0.48 | 0.96 |
| MAX | 0.54 | 1.23 |

b)

| Sample | r (1:2) | r (3:2) |
|---|---|---|
| OMH-1 | 0.54 | 0.93 |
| OMH-2 | 0.45 | 0.92 |
| OMH-3 | 0.47 | 0.95 |
| OMH-4 | 0.44 | 0.9 |
| OMH-5 | 0.50 | 1.12 |
| OMH-6 | 0.48 | 1.1 |
| MIN | 0.44 | 0.90 |
| MAX | 0.54 | 1.12 |

**Table S6-** Signal intensity ratio calculated on BLH spectra recorded at 500MHz (a) and 600MHz (b)

a)

| Sample | r (1:2) | r (3:2) |
|---|---|---|
| BLH-1 | 0.29 | 0.18 |
| BLH-2 | 0.34 | 0.31 |
| BLH-3 | 0.34 | 0.32 |
| BLH-4 | 0.38 | 0.4 |
| BLH-5 | 0.36 | 0.36 |
| BLH-6 | 0.38 | 0.38 |
| BLH-7 | 0.37 | 0.4 |
| MIN | 0.29 | 0.18 |
| MAX | 0.38 | 0.40 |

b)

| Sample | r (1:2) | r (3:2) |
|---|---|---|
| BLH-1 | 0.27 | 0.16 |
| BLH-2 | 0.31 | 0.29 |
| BLH-3 | 0.32 | 0.28 |
| BLH-4 | 0.35 | 0.37 |
| BLH-5 | 0.33 | 0.33 |
| BLH-6 | 0.35 | 0.35 |
| BLH-7 | 0.33 | 0.38 |
| MIN | 0.27 | 0.16 |
| MAX | 0.35 | 0.38 |

**Table S7 -** Signal ratios measured on spectra recorded with 16 or 8 scans. Six samples of the same PMH batch were prepared. S/N was calculated for the most intense peak of the 3.75-4.55 ppm region.

| **NS 16** | **r(1:2)** | **r(3:2)** | **S/N** |
|---|---|---|---|
| PMH A | 0.74 | 2.22 | 3458 |
| PMH B | 0.74 | 2.23 | 3447 |
| PMH C | 0.81 | 2.01 | 3369 |
| PMH D | 0.74 | 2.22 | 3392 |
| PMH E | 0.74 | 2.21 | 3321 |
| PMH F | 0.74 | 2.22 | 3328 |
| mean | 0.754 | 2.187 | 3385.8 |
| Std | 0.030 | 0.087 | 58.02 |
| CV% | 3.9 | 4.0 | 1.7 |
| | | | |
| **NS 8** | **r(1:2)** | **r(3:2)** | |
| PMH A | 0.75 | 2.23 | 2451 |
| PMH B | 0.75 | 2.22 | 2478 |
| PMH C | 0.82 | 2.01 | 2424 |
| PMH D | 0.75 | 2.16 | 2387 |
| PMH E | 0.75 | 2.21 | 2333 |
| PMH F | 0.74 | 2.23 | 2376 |
| mean | 0.758 | 2.177 | 2408.2 |
| Std | 0.029 | 0.085 | 53.1 |
| CV% | 3.8 | 3.9 | 2.2 |

**Table S8 -** Signal intensity ratio r (1:2) calculated on BMH spectra recorded in two different laboratories

| **Sample** | **r(1:2) Lab 1** | **r(1:2) Lab 2** |
|---|---|---|
| E1 | 1.53 | 1.56 |
| F1 | 1.51 | 1.55 |
| G1 | 1.48 | 1.43 |
| H1 | 1.40 | 1.43 |
| E2 | 1.73 | 1.78 |
| F2 | 1.78 | 1.75 |
| G2 | 1.86 | 1.85 |
| K1 | 1.60 | 1.59 |
| L1 | 1.48 | 1.41 |
| M1 | 1.43 | 1.42 |
| N1 | 1.38 | 1.20 |
| O1 | 1.41 | 1.40 |
| P1 | 1.48 | 1.50 |
| Q1 | 1.52 | 1.47 |
| R1 | 1.53 | 1.53 |
| S1 | 1.60 | 1.78 |
| O2 | 1.65 | 1.65 |
| P2 | 1.64 | 1.68 |
| Q2 | 1.62 | 1.58 |
| R2 | 1.62 | 1.41 |
| **min** | 1.38 | 1.20 |
| **max** | 1.86 | 1.85 |

**Table S9 – Proton and carbon chemical shifts of signals integrated of the HSQC spectrum**

| Fragment | ν(F2) [ppm] | ν(F1) [ppm] |
|---|---|---|
| $^{2}A_{NS,6X}$ | 3.31 | 61.2 |
| $^{2}A_{NS,3S,6X}$ | 3.44 | 59.4 |
| $^{2}A_{NAc,6X}$ | 3.92 | 56.4 |
| $^{2}A_{NH2,6X}$ | 3.92 | 56.9 |
| $^{6}A_{NY,3X}$ | 3.87 | 62.6 |
| $^{6}A_{NY,3X,6S}$ | 4.36 | 69.1 |
| $^{1}A_{NS,6X}$-(G) | 5.59 | 100.3 |
| $^{1}A_{NS,3S,6X}$ | 99.0 | 5.52 |
| $^{1}A_{NS,6X}$-($I_{2S}$) + $^{1}A_{NAc}$-(G) | 99.8 | 5.37 |
| $^{1}A_{NS,6X}$-(I) | 983 | 5.36 |
| $^{1}A_{NAc,6x}$-($I_{2x}$) | 96.5 | 5.15 |
| $^{1}A_{NS,6X}$-aRed + $^{1}A_{NH2,6X}$ | 93.9 | 5.45 |
| $^{1}A_{NAc,6X}$-aRed | 93.4 | 5.21 |
| $^{1}A_{NS,6X}$-(GalA) + $^{1}$Unk | 102.0 | 5.4 |
| $^{1}I_{2S}$-($A_{NY',3X,6X}$) + $^{1}$GalA | 102.3 | 5.20 |
| $^{1}$I-($A_{NY',6S}$) | 104.9 | 5.02 |
| $^{1}$I-($A_{NY'}$) | 104.7 | 4.95 |
| $^{1}G_{2S}$ | 103.7 | 4.70 |
| $^{1}$G-($A_{NS,3S,6X}$) | 103.9 | 4.62 |
| $^{1}$G-($A_{NS,6X}$) | 104.9 | 4.62 |
| $^{1}$G-($A_{NAc,6X}$) | 105.1 | 4.51 |
| $^{1}$Gal-(Xyl) | 104.3 | 4.54 |
| $^{1}$Xyl-(Ser) | 105.2 | 4.45 |
| $^{1}$Xyl-(ox-Ser) | 105.8 | 4.45 |
| $^{1}$G-(Gal) + $^{1}$Gal-(Gal) | 106.7 | 4.66 |
| $^{5}$GalA | 74.5 | 4.70 |
| $^{1}$2,3-epoxide | 97.5 | 5.43 |
| $^{1}I_{2S}$-($A_{NH2,6X}$) | 101.3 | 5.25 |
| $^{4}G_{NR}$ | 74.8 | 3.55 |
| $^{3}G_{NR}$ | 78.0 | 3.54 |
| $^{2}A_{NAc}$-ox | 59.0 | 4.37 |

## **Table S10 -**Molar percentual of glucosamine residues of 39 porcine heparin samples

| Samples | ANH2,6x | ANAc,6x | ANS,3S,6x | ANS,6X | ANAc,6X-G | ANAc,6X-I | ANS,6X-G | ANS,6X-I | ANS,6X-I2S | ANS,6X-GalA | ANS,6X-Epox | ANAc,6XaRed | ANS,6XaRed | % 6S |
|---|---|---|---|---|---|---|---|---|---|---|---|---|---|---|
| PMH-1 | < LOD | 11.5 | 5.5 | 83.0 | 11.5 | < LOD | 9.0 | 9.9 | 59.1 | 4.3 | < LOD | < LOD | 0.8 | 81.1 |
| PMH-2 | < LOD | 13.2 | 5.4 | 81.4 | 12.6 | 0.6 | 9.9 | 8.8 | 62.0 | 0.6 | < LOD | < LOD | < LOD | 79.1 |
| PMH-3 | < LOD | 13.7 | 5.6 | 79.9 | 13.7 | < LOD | 9.9 | 7.7 | 60.5 | 1.3 | < LOD | < LOD | < LOD | 79.8 |
| PMH-4 | < LOD | 13.2 | 5.1 | 81.7 | 13.2 | < LOD | 10.1 | 8.7 | 59.6 | 3.2 | < LOD | < LOD | < LOD | 79.0 |
| PMH-5 | < LOD | 11.8 | 4.4 | 83.8 | 11.8 | < LOD | 9.7 | 8.5 | 63.4 | 2.2 | < LOD | < LOD | < LOD | 78.7 |
| PMH-6 | 1.5 | 14.7 | 4.9 | 78.9 | 14.7 | < LOD | 9.5 | 8.8 | 59.9 | 1.0 | < LOD | < LOD | < LOD | 75.7 |
| PMH-7 | 1.5 | 12.6 | 5.0 | 80.9 | 10.9 | 0.8 | 9.0 | 8.8 | 62.9 | < LOD | < LOD | 0.9 | < LOD | 77.8 |
| PMH-8 | 1.0 | 12.9 | 5.5 | 80.6 | 12.9 | < LOD | 9.9 | 7.6 | 61.5 | 1.6 | < LOD | < LOD | < LOD | 77.4 |
| PMH-9 | 1.6 | 13.3 | 4.8 | 80.3 | 12.3 | 1.1 | 9.2 | 8.2 | 62.8 | < LOD | < LOD | < LOD | < LOD | 77.1 |
| PMH-10 | 1.6 | 14.3 | 4.9 | 79.3 | 14.3 | < LOD | 9.5 | 8.5 | 61.1 | < LOD | < LOD | < LOD | < LOD | 76.5 |
| PMH-11 | 2.2 | 12.5 | 4.7 | 80.5 | 11.8 | < LOD | 10.4 | 9.0 | 60.0 | 2.3 | 1.0 | 0.8 | < LOD | 76.7 |
| PMH-12 | 1.6 | 11.8 | 4.7 | 82.0 | 11.0 | 0.7 | 10.7 | 7.9 | 62.8 | 0.7 | < LOD | < LOD | < LOD | 78.5 |
| PMH-13 | 1.9 | 14.0 | 4.5 | 79.6 | 13.2 | 0.8 | 9.5 | 8.3 | 60.0 | 1.2 | < LOD | < LOD | < LOD | 76.9 |
| PMH-14 | 1.5 | 13.4 | 4.8 | 80.4 | 13.4 | < LOD | 10.4 | 8.6 | 60.2 | 1.0 | < LOD | < LOD | < LOD | 77.4 |
| PMH-15 | 2.8 | 13.4 | 4.5 | 79.2 | 13.4 | < LOD | 8.5 | 8.8 | 63.2 | < LOD | < LOD | < LOD | < LOD | 76.3 |
| PMH-16 | 1.4 | 13.9 | 4.6 | 80.2 | 13.9 | < LOD | 9.2 | 8.5 | 61.0 | 1.2 | < LOD | < LOD | < LOD | 76.1 |
| PMH-17 | 2.1 | 12.8 | 5.2 | 80.0 | 12.8 | < LOD | 8.0 | 7.6 | 63.9 | 1.4 | < LOD | < LOD | < LOD | 76.8 |
| PMH-18 | 1.8 | 15.1 | 5.0 | 78.2 | 14.3 | 0.8 | 8.3 | 8.8 | 61.1 | < LOD | < LOD | < LOD | < LOD | 75.0 |
| PMH-19 | 1.7 | 11.5 | 5.3 | 81.5 | 11.5 | < LOD | 8.8 | 8.0 | 63.8 | 1.1 | < LOD | < LOD | < LOD | 79.6 |
| PMH-20 | 1.9 | 13.6 | 5.7 | 78.9 | 13.6 | < LOD | 8.9 | 6.7 | 62.7 | < LOD | < LOD | < LOD | < LOD | 78.4 |
| PMH-21 | 0.7 | 12.9 | 4.8 | 81.6 | 12.9 | < LOD | 10.1 | 9.4 | 61.7 | < LOD | < LOD | < LOD | < LOD | 77.4 |
| PMH-22 | < LOD | 11.4 | 4.9 | 83.8 | 10.8 | 0.6 | 9.9 | 8.4 | 63.3 | 0.8 | < LOD | < LOD | 1.3 | 77.5 |
| PMH-23 | 1.6 | 13.9 | 4.8 | 79.7 | 13.0 | 0.9 | 9.4 | 9.0 | 60.5 | < LOD | < LOD | < LOD | 0.8 | 74.7 |
| PMH-24 | 1.2 | 13.7 | 4.1 | 81.1 | 11.5 | 0.6 | 10.3 | 8.7 | 59.0 | 2.0 | < LOD | 1.6 | 1.2 | 73.6 |
| PMH-25 | 2.0 | 12.5 | 5.0 | 80.5 | 11.5 | 0.9 | 9.9 | 6.5 | 62.9 | 0.7 | < LOD | < LOD | < LOD | 77.1 |
| PMH-26 | 0.6 | 12.3 | 5.0 | 82.1 | 11.6 | 0.7 | 10.3 | 6.9 | 63.7 | 0.8 | < LOD | < LOD | < LOD | 77.1 |
| PMH-27 | 0.7 | 13.2 | 5.3 | 80.8 | 13.2 | < LOD | 9.7 | 8.1 | 62.0 | 0.6 | < LOD | < LOD | < LOD | 78.3 |
| PMH-28 | 0.9 | 11.7 | 4.7 | 82.7 | 10.7 | 1.0 | 10.1 | 8.7 | 62.6 | 0.9 | < LOD | < LOD | < LOD | 76.7 |
| PMH-29 | 1.0 | 13.7 | 4.8 | 80.5 | 11.8 | 0.8 | 9.9 | 8.5 | 63.1 | < LOD | < LOD | 1.0 | < LOD | 76.1 |
| PMH-30 | 1.7 | 11.5 | 6.7 | 80.1 | 10.9 | 0.6 | 9.1 | 7.6 | 62.2 | 1.0 | < LOD | < LOD | < LOD | 79.3 |
| PMH-31 | 1.6 | 12.2 | 5.7 | 80.6 | 11.4 | 0.8 | 9.2 | 8.4 | 61.6 | 1.4 | < LOD | < LOD | < LOD | 79.2 |
| PMH-32 | 1.7 | 15.3 | 5.4 | 77.6 | 14.0 | 0.7 | 9.3 | 8.5 | 58.1 | 1.6 | < LOD | 0.7 | < LOD | 74.7 |
| PMH-33 | 2.0 | 14.7 | 4.9 | 78.5 | 13.7 | 0.9 | 9.0 | 9.0 | 59.5 | 0.6 | < LOD | < LOD | < LOD | 77.3 |
| PMH-34 | 2.4 | 10.0 | 5.4 | 82.2 | 9.3 | 0.7 | 9.0 | 8.1 | 63.9 | 1.2 | < LOD | < LOD | < LOD | 81.2 |
| PMH-35 | 1.0 | 11.3 | 5.2 | 82.6 | 11.3 | < LOD | 9.4 | 9.3 | 60.0 | 2.5 | 0.8 | < LOD | 0.8 | 82.3 |
| PMH-36 | 1.3 | 11.8 | 4.1 | 82.8 | 11.8 | < LOD | 9.9 | 7.9 | 61.0 | 3.4 | < LOD | < LOD | 0.7 | 76.8 |
| PMH-37 | 1.3 | 11.2 | 4.1 | 83.4 | 10.0 | 0.6 | 9.8 | 9.2 | 60.3 | 3.6 | < LOD | 0.6 | < LOD | 77.1 |
| PMH-38 | 1.1 | 11.1 | 3.8 | 84.0 | 11.1 | < LOD | 11.0 | 7.0 | 62.3 | 3.2 | < LOD | < LOD | < LOD | 76.7 |
| PMH-39 | 2.0 | 12.8 | 5.1 | 80.1 | 12.2 | 0.6 | 9.3 | 8.5 | 61.8 | 0.6 | < LOD | < LOD | < LOD | 78.4 |

**Table S11-** Molar percentual of uronic acids residues of 39 porcine mucosa heparin samples

| Samples | G2OH | G2S | I2OH | I2S | G-ANAc,6X | G-ANS,6X | G-ANS,3S,6X | I-ANY | I-ANY,6S | I2S-ANH2,6X | I2S-ANY,3X,6X | GalA | Epox |
|---|---|---|---|---|---|---|---|---|---|---|---|---|---|
| PMH-1 | 15.2 | < LOD | 8.8 | 72.7 | 5.4 | 6.4 | 3.4 | 2.2 | 6.6 | < LOD | 72.7 | 3.3 | < LOD |
| PMH-2 | 15.5 | < LOD | 9.3 | 75.2 | 6.4 | 6.7 | 2.4 | 2.7 | 6.6 | < LOD | 75.2 | < LOD | < LOD |
| PMH-3 | 14.8 | < LOD | 9.4 | 75.8 | 5.4 | 6.1 | 3.3 | 2.7 | 6.7 | 1.5 | 74.3 | < LOD | < LOD |
| PMH-4 | 16.1 | < LOD | 8.9 | 75.0 | 7.1 | 6.4 | 2.6 | 2.6 | 6.3 | < LOD | 75.0 | < LOD | < LOD |
| PMH-5 | 15.1 | < LOD | 8.9 | 75.3 | 6.3 | 6.2 | 2.6 | 2.2 | 6.6 | < LOD | 74.6 | 0.8 | < LOD |
| PMH-6 | 15.9 | < LOD | 9.2 | 74.9 | 6.8 | 6.4 | 2.7 | 2.6 | 6.6 | 1.6 | 73.4 | < LOD | < LOD |
| PMH-7 | 14.0 | < LOD | 9.9 | 76.2 | 5.3 | 5.8 | 2.9 | 3.2 | 6.7 | 1.9 | 74.3 | < LOD | < LOD |
| PMH-8 | 15.8 | < LOD | 8.7 | 75.5 | 7.0 | 6.0 | 2.8 | 3.0 | 5.7 | 1.4 | 74.2 | < LOD | < LOD |
| PMH-9 | 14.9 | < LOD | 9.4 | 75.8 | 5.8 | 6.6 | 2.5 | 2.6 | 6.8 | 1.7 | 74.1 | < LOD | < LOD |
| PMH-10 | 14.8 | < LOD | 8.6 | 76.5 | 6.3 | 5.8 | 2.7 | 2.5 | 6.2 | 1.7 | 74.8 | < LOD | < LOD |
| PMH-11 | 16.3 | < LOD | 9.2 | 72.6 | 7.1 | 6.9 | 2.3 | 2.7 | 6.5 | 1.5 | 71.1 | 0.9 | 1.0 |
| PMH-12 | 14.7 | < LOD | 7.7 | 77.7 | 5.4 | 6.7 | 2.5 | 2.3 | 5.4 | 1.8 | 75.9 | < LOD | < LOD |
| PMH-13 | 14.8 | < LOD | 9.8 | 75.4 | 5.9 | 5.6 | 3.3 | 2.8 | 7.0 | 2.2 | 73.2 | < LOD | < LOD |
| PMH-14 | 15.4 | < LOD | 9.4 | 75.2 | 6.3 | 6.5 | 2.6 | 2.7 | 6.7 | 1.6 | 73.6 | < LOD | < LOD |
| PMH-15 | 14.1 | < LOD | 10.0 | 75.9 | 5.7 | 5.8 | 2.6 | 3.1 | 6.9 | 2.1 | 73.7 | < LOD | < LOD |
| PMH-16 | 15.9 | < LOD | 9.7 | 74.4 | 6.9 | 6.3 | 2.7 | 3.2 | 6.5 | 1.8 | 72.7 | < LOD | < LOD |
| PMH-17 | 15.1 | < LOD | 9.2 | 75.7 | 5.6 | 6.6 | 2.9 | 2.5 | 6.7 | 2.0 | 73.7 | < LOD | < LOD |
| PMH-18 | 16.4 | < LOD | 9.7 | 73.9 | 6.1 | 6.9 | 3.4 | 3.2 | 6.6 | 1.7 | 72.2 | < LOD | < LOD |
| PMH-19 | 14.4 | < LOD | 7.9 | 77.7 | 6.5 | 5.3 | 2.7 | 2.4 | 5.5 | 1.7 | 76.0 | < LOD | < LOD |
| PMH-20 | 13.1 | < LOD | 9.2 | 77.8 | 4.6 | 5.1 | 3.4 | 3.4 | 5.8 | 2.2 | 75.6 | < LOD | < LOD |
| PMH-21 | 15.4 | < LOD | 7.7 | 77.0 | 5.9 | 6.8 | 2.7 | 2.4 | 5.3 | 1.1 | 75.9 | < LOD | < LOD |
| PMH-22 | 12.9 | < LOD | 7.5 | 79.6 | 4.4 | 6.3 | 2.1 | 2.4 | 5.1 | 1.0 | 78.7 | < LOD | < LOD |
| PMH-23 | 16.1 | < LOD | 8.7 | 75.2 | 6.5 | 7.1 | 2.6 | 2.4 | 6.3 | 2.3 | 72.9 | < LOD | < LOD |
| PMH-24 | 16.7 | < LOD | 9.7 | 73.1 | 7.2 | 6.6 | 2.8 | 3.2 | 6.5 | 1.6 | 71.5 | 0.6 | < LOD |
| PMH-25 | 13.3 | < LOD | 9.4 | 77.3 | 4.6 | 5.8 | 2.9 | 3.1 | 6.3 | 2.0 | 75.3 | < LOD | < LOD |
| PMH-26 | 15.3 | < LOD | 7.9 | 76.8 | 6.7 | 5.8 | 2.8 | 2.7 | 5.2 | 0.9 | 75.9 | < LOD | < LOD |
| PMH-27 | 14.5 | < LOD | 7.9 | 77.6 | 6.0 | 6.2 | 2.3 | 2.7 | 5.2 | 0.7 | 76.9 | < LOD | < LOD |
| PMH-28 | 14.9 | < LOD | 7.4 | 77.7 | 6.0 | 6.6 | 2.3 | 2.2 | 5.2 | 1.3 | 76.4 | < LOD | < LOD |
| PMH-29 | 13.4 | < LOD | 9.2 | 77.4 | 4.8 | 6.8 | 1.9 | 2.6 | 6.6 | 1.0 | 76.4 | < LOD | < LOD |
| PMH-30 | 14.9 | < LOD | 7.3 | 77.8 | 5.6 | 6.5 | 2.8 | 2.1 | 5.2 | 1.5 | 76.3 | < LOD | < LOD |
| PMH-31 | 14.4 | < LOD | 7.3 | 78.3 | 6.4 | 5.4 | 2.5 | 2.3 | 4.9 | 1.6 | 76.7 | < LOD | < LOD |
| PMH-32 | 17.5 | < LOD | 9.3 | 73.2 | 7.9 | 6.6 | 3.0 | 2.7 | 6.6 | 1.7 | 71.6 | < LOD | < LOD |
| PMH-33 | 16.5 | < LOD | 9.4 | 74.1 | 6.6 | 7.0 | 3.0 | 2.6 | 6.8 | 2.0 | 72.1 | < LOD | < LOD |
| PMH-34 | 13.6 | < LOD | 8.3 | 78.1 | 4.6 | 6.5 | 2.6 | 2.6 | 5.7 | 2.2 | 75.9 | < LOD | < LOD |
| PMH-35 | 13.0 | < LOD | 7.8 | 77.3 | 5.0 | 5.4 | 2.6 | 2.1 | 5.7 | 1.4 | 75.9 | 1.2 | 0.7 |
| PMH-36 | 14.5 | < LOD | 7.1 | 77.6 | 6.1 | 6.2 | 2.3 | 2.5 | 4.6 | 1.4 | 76.2 | 0.8 | < LOD |
| PMH-37 | 14.8 | < LOD | 7.3 | 76.5 | 5.7 | 6.9 | 2.2 | 2.3 | 5.0 | 1.4 | 75.1 | 1.4 | < LOD |
| PMH-38 | 15.2 | < LOD | 7.4 | 77.4 | 6.6 | 6.7 | 2.0 | 2.5 | 4.9 | 1.1 | 76.4 | < LOD | < LOD |
| PMH-39 | 15.1 | < LOD | 9.0 | 76.0 | 6.0 | 6.3 | 2.8 | 2.1 | 6.8 | 2.1 | 73.9 | < LOD | < LOD |

**Table S12 -** Linkage region and sulfation degree of 39 porcine mucosa heparin samples

| Samples | LR | %ox Ser | SDEG |
|---|---|---|---|
| PMH-1 | 3.1 | 59.0 | 2.48 |
| PMH-2 | 3.8 | 70.0 | 2.46 |
| PMH-3 | 4.3 | 66.4 | 2.47 |
| PMH-4 | 3.8 | 79.7 | 2.46 |
| PMH-5 | 3.8 | 55.1 | 2.47 |
| PMH-6 | 5.1 | 100.0 | 2.39 |
| PMH-7 | 4.0 | 0.0 | 2.45 |
| PMH-8 | 3.8 | 100.0 | 2.44 |
| PMH-9 | 6.2 | 0.0 | 2.43 |
| PMH-10 | 5.3 | 0.0 | 2.42 |
| PMH-11 | 3.9 | 0.0 | 2.39 |
| PMH-12 | 4.1 | 47.3 | 2.48 |
| PMH-13 | 5.5 | 0.0 | 2.41 |
| PMH-14 | 4.9 | 0.0 | 2.43 |
| PMH-15 | 6.4 | 0.0 | 2.40 |
| PMH-16 | 5.2 | 24.0 | 2.40 |
| PMH-17 | 6.0 | 74.0 | 2.43 |
| PMH-18 | 6.2 | 100.0 | 2.37 |
| PMH-19 | 3.5 | 100.0 | 2.49 |
| PMH-20 | 6.1 | 0.0 | 2.46 |
| PMH-21 | 2.3 | 57.6 | 2.45 |
| PMH-22 | 1.4 | 0.0 | 2.51 |
| PMH-23 | 5.2 | 100.0 | 2.39 |
| PMH-24 | 3.2 | 36.0 | 2.36 |
| PMH-25 | 5.9 | 0.0 | 2.45 |
| PMH-26 | 2.1 | 100.0 | 2.46 |
| PMH-27 | 2.7 | 100.0 | 2.47 |
| PMH-28 | 2.2 | 52.2 | 2.47 |
| PMH-29 | 5.7 | 36.7 | 2.44 |
| PMH-30 | 2.8 | 100.0 | 2.51 |
| PMH-31 | 3.3 | 100.0 | 2.49 |
| PMH-32 | 5.0 | 79.8 | 2.36 |
| PMH-33 | 5.2 | 100.0 | 2.40 |
| PMH-34 | 4.3 | 0.0 | 2.52 |
| PMH-35 | 2.5 | 100.0 | 2.53 |
| PMH-36 | 3.6 | 100.0 | 2.45 |
| PMH-37 | 2.6 | 76.5 | 2.45 |
| PMH-38 | 3.7 | 70.0 | 2.46 |
| PMH-39 | 4.6 | 100.0 | 2.45 |

**Table S13 -** Molar percentual of glucosamine residues of 39 bovine mucosa heparin samples

| Sample | ANH2,6x | ANAc,6x | ANS,3S,6x | ANS,6X | ANAc,6X-G | ANAc,6X-I | ANS,6X-G | ANS,6X-I | ANS,6X-I2S | ANS,6X-GalA | ANS,6X-Epox | ANAc,6XaRed | ANS,6XaRed | % 6S |
|---|---|---|---|---|---|---|---|---|---|---|---|---|---|---|
| BMH-1 | < LOD | 6.8 | 2.1 | 91.2 | 6.8 | < LOD | 17.2 | 4.2 | 67.4 | 1.0 | < LOD | < LOD | 1.4 | 49.6 |
| BMH-2 | < LOD | 7.2 | 1.3 | 91.6 | 7.2 | < LOD | 17.1 | 3.2 | 70.2 | < LOD | < LOD | < LOD | 1.1 | 51.2 |
| BMH-3 | 0.6 | 5.8 | 1.1 | 92.5 | 5.3 | < LOD | 15.3 | 3.6 | 73.3 | < LOD | < LOD | 0.5 | < LOD | 50.6 |
| BMH-4 | < LOD | 6.0 | 1.6 | 92.4 | 6.0 | < LOD | 15.3 | 3.9 | 73.2 | < LOD | < LOD | < LOD | < LOD | 49.8 |
| BMH-5 | 0.8 | 6.3 | 1.3 | 91.5 | 6.3 | < LOD | 15.4 | 3.8 | 71.1 | 0.6 | < LOD | < LOD | 0.6 | 50.9 |
| E1 | < LOD | 8.7 | 1.9 | 89.5 | 8.7 | < LOD | 15.9 | 4.4 | 67.2 | 2.0 | < LOD | < LOD | < LOD | 52.5 |
| F1 | < LOD | 7.5 | 1.4 | 91.0 | 6.6 | < LOD | 17.3 | 5.3 | 67.0 | 1.5 | < LOD | 1.0 | < LOD | 52.0 |
| G1 | < LOD | 7.0 | 1.8 | 91.2 | 5.9 | < LOD | 15.5 | 4.1 | 69.8 | 1.2 | < LOD | 1.1 | 0.6 | 53.1 |
| H1 | < LOD | 8.0 | 1.8 | 90.2 | 7.1 | < LOD | 15.8 | 4.9 | 68.5 | 0.9 | < LOD | 0.9 | < LOD | 54.8 |
| E2 | < LOD | 11.3 | 2.0 | 86.8 | 10.5 | < LOD | 16.4 | 4.7 | 63.9 | < LOD | < LOD | 0.8 | 1.8 | 49.2 |
| F2 | 1.8 | 11.0 | 1.8 | 85.5 | 10.3 | < LOD | 16.5 | 5.0 | 63.1 | 0.8 | < LOD | 0.7 | < LOD | 49.8 |
| G2 | 1.0 | 11.8 | 1.5 | 85.6 | 11.0 | < LOD | 16.3 | 5.2 | 61.9 | 1.0 | < LOD | 0.8 | 1.2 | 48.1 |
| K1 | 0.8 | 8.0 | 1.3 | 89.9 | 8.0 | < LOD | 15.5 | 3.8 | 70.1 | 0.5 | < LOD | < LOD | < LOD | 50.4 |
| L1 | 1.3 | 6.4 | 1.6 | 90.7 | 5.7 | < LOD | 16.1 | 4.9 | 68.3 | 1.4 | < LOD | 0.7 | < LOD | 52.3 |
| M1 | < LOD | 5.9 | 1.9 | 92.2 | 5.9 | < LOD | 16.8 | 3.5 | 68.8 | 1.3 | < LOD | < LOD | 1.7 | 52.7 |
| N1 | 0.8 | 5.6 | 2.4 | 91.3 | 5.0 | < LOD | 15.7 | 3.9 | 70.0 | 0.6 | < LOD | 0.6 | 1.2 | 54.1 |
| O1 | 0.9 | 5.5 | 1.6 | 92.0 | 4.9 | < LOD | 15.6 | 3.4 | 71.4 | 0.7 | < LOD | 0.7 | 0.9 | 54.0 |
| P1 | < LOD | 5.8 | 1.4 | 92.8 | 5.8 | < LOD | 14.9 | 3.1 | 72.9 | 0.6 | < LOD | < LOD | 1.4 | 50.4 |
| Q1 | 1.1 | 7.2 | 1.1 | 90.6 | 7.2 | < LOD | 17.0 | 2.9 | 70.5 | < LOD | < LOD | < LOD | < LOD | 50.9 |
| R1 | 0.7 | 6.7 | 1.7 | 91.0 | 6.7 | < LOD | 15.7 | 3.8 | 69.7 | 1.2 | < LOD | < LOD | < LOD | 51.1 |
| S1 | < LOD | 6.2 | 1.6 | 92.2 | 6.2 | < LOD | 15.9 | 3.3 | 71.3 | < LOD | < LOD | < LOD | 1.7 | 48.8 |
| O2 | < LOD | 6.1 | 2.0 | 91.3 | 6.1 | < LOD | 17.0 | 4.7 | 58.1 | 8.9 | 1.3 | < LOD | 1.3 | 50.5 |
| P2 | < LOD | 6.0 | 1.1 | 92.9 | 6.0 | < LOD | 17.6 | 4.4 | 59.4 | 8.1 | 1.5 | < LOD | 2.0 | 49.8 |
| Q2 | < LOD | 5.1 | 1.4 | 92.9 | 5.1 | < LOD | 17.2 | 4.1 | 60.5 | 8.7 | 1.2 | < LOD | 1.3 | 50.5 |
| R2 | 1.4 | 5.5 | 2.0 | 91.1 | 5.5 | < LOD | 16.4 | 5.1 | 51.0 | 13.7 | 5.1 | < LOD | < LOD | 51.2 |
| BMH-6 | < LOD | 6.7 | 2.9 | 90.4 | 5.8 | < LOD | 16.4 | 5.1 | 66.8 | 0.8 | < LOD | 0.9 | 1.2 | 54.6 |
| BMH-7 | 1.3 | 8.8 | 2.0 | 87.9 | 8.8 | < LOD | 15.1 | 5.0 | 66.4 | 1.4 | < LOD | < LOD | < LOD | 52.4 |
| BMH-8 | 1.0 | 7.3 | 1.8 | 90.0 | 7.3 | < LOD | 15.4 | 4.3 | 68.1 | 1.8 | < LOD | < LOD | < LOD | 53.5 |
| BMH-9 | < LOD | 7.6 | 2.4 | 90.1 | 6.6 | < LOD | 15.9 | 4.2 | 66.3 | 2.4 | < LOD | 1.0 | 1.3 | 50.2 |
| BMH-10 | 1.1 | 9.0 | 2.0 | 87.9 | 9.0 | < LOD | 15.9 | 4.6 | 65.8 | 1.8 | < LOD | < LOD | < LOD | 49.7 |
| BMH-11 | < LOD | 8.6 | 1.2 | 90.3 | 8.6 | < LOD | 17.0 | 3.7 | 67.0 | 1.2 | < LOD | < LOD | 1.4 | 47.1 |
| BMH-12 | 0.7 | 6.0 | 1.7 | 91.6 | 5.1 | < LOD | 15.2 | 4.4 | 65.7 | 5.7 | < LOD | 0.9 | 0.6 | 54.9 |
| BMH-13 | < LOD | 6.9 | 1.8 | 91.3 | 6.1 | < LOD | 15.1 | 4.8 | 64.4 | 5.8 | < LOD | 0.8 | 1.1 | 53.4 |
| BMH-14 | < LOD | 7.5 | 2.3 | 90.3 | 6.6 | < LOD | 14.7 | 4.5 | 64.8 | 4.9 | < LOD | 0.9 | 1.4 | 55.0 |
| BMH-15 | 0.8 | 7.8 | 2.3 | 89.2 | 7.8 | < LOD | 16.3 | 3.8 | 63.4 | 6.5 | < LOD | < LOD | < LOD | 55.8 |
| BMH-16 | < LOD | 7.4 | 2.2 | 90.5 | 7.4 | < LOD | 14.5 | 4.1 | 70.7 | < LOD | < LOD | < LOD | 1.1 | 56.5 |
| BMH-17 | < LOD | 8.0 | 2.1 | 89.9 | 8.0 | < LOD | 14.9 | 4.9 | 65.3 | 3.2 | < LOD | < LOD | 1.5 | 54.9 |
| BMH-18 | < LOD | 7.4 | 2.1 | 90.6 | 6.7 | < LOD | 14.9 | 4.5 | 65.5 | 4.4 | < LOD | 0.6 | 1.4 | 58.0 |
| BMH-19 | 0.6 | 7.4 | 2.1 | 90.0 | 6.7 | < LOD | 14.6 | 4.9 | 63.7 | 5.5 | < LOD | 0.7 | 1.1 | 55.5 |

**Table S14 -** Molar percentual of uronic acid residues of 39 bovine mucosa heparin samples

| Sample | G2OH | G2S | I2OH | I2S | G-ANAc,6X | G-ANS,6X | G-ANS,3S,6X | I-ANY | I-ANY,6S | I2S-ANH2,6X | I2S-ANY,3X,6X | GalA | Epox |
|---|---|---|---|---|---|---|---|---|---|---|---|---|---|
| BMH-1 | 13.5 | 1.9 | 4.7 | 77.9 | 7.9 | 5.6 | < LOD | 3.1 | 1.6 | 0.8 | 77.1 | 2.0 | < LOD |
| BMH-2 | 13.8 | 1.9 | 4.5 | 79.8 | 8.7 | 5.0 | < LOD | 3.3 | 1.2 | 1.6 | 78.3 | < LOD | < LOD |
| BMH-3 | 13.7 | 2.1 | 3.8 | 80.4 | 8.0 | 4.9 | 0.8 | 2.9 | 0.9 | 1.2 | 79.2 | < LOD | < LOD |
| BMH-4 | 12.5 | 1.8 | 3.8 | 81.9 | 8.7 | 3.8 | < LOD | 2.8 | 1.0 | 1.3 | 80.6 | < LOD | < LOD |
| BMH-5 | 12.4 | 1.6 | 3.5 | 82.5 | 8.0 | 4.4 | < LOD | 2.7 | 0.9 | 1.6 | 80.9 | < LOD | < LOD |
| E1 | 14.3 | 2.0 | 5.2 | 78.5 | 8.6 | 5.7 | < LOD | 3.2 | 2.1 | 0.8 | 77.7 | < LOD | < LOD |
| F1 | 13.6 | 1.8 | 4.4 | 80.2 | 8.2 | 5.4 | < LOD | 2.8 | 1.6 | < LOD | 80.2 | < LOD | < LOD |
| G1 | 13.5 | 2.1 | 4.9 | 79.5 | 8.5 | 4.9 | < LOD | 3.1 | 1.8 | < LOD | 79.5 | < LOD | < LOD |
| H1 | 13.3 | 1.9 | 5.1 | 79.8 | 6.9 | 5.8 | < LOD | 3.0 | 2.1 | 0.6 | 79.2 | < LOD | < LOD |
| E2 | 17.6 | 1.8 | 6.1 | 74.5 | 10.5 | 7.0 | < LOD | 3.7 | 2.4 | 1.2 | 73.4 | < LOD | < LOD |
| F2 | 17.8 | 1.8 | 5.7 | 74.7 | 11.3 | 5.7 | 0.8 | 3.7 | 1.9 | 1.6 | 73.1 | < LOD | < LOD |
| G2 | 17.9 | 1.9 | 6.2 | 74.0 | 11.7 | 6.3 | < LOD | 4.1 | 2.1 | 1.3 | 72.7 | < LOD | < LOD |
| K1 | 13.8 | 1.6 | 4.1 | 80.6 | 8.7 | 5.0 | < LOD | 2.9 | 1.2 | 1.2 | 79.3 | < LOD | < LOD |
| L1 | 12.9 | 2.1 | 4.4 | 80.7 | 7.6 | 5.2 | < LOD | 2.9 | 1.4 | 1.4 | 79.3 | < LOD | < LOD |
| M1 | 13.6 | 1.7 | 4.0 | 80.6 | 7.2 | 5.7 | 0.8 | 2.5 | 1.6 | 1.5 | 79.1 | < LOD | < LOD |
| N1 | 12.9 | 1.7 | 3.9 | 81.6 | 6.9 | 5.4 | 0.5 | 3.0 | 0.9 | 1.6 | 79.9 | < LOD | < LOD |
| O1 | 12.8 | 2.0 | 3.7 | 81.5 | 7.2 | 4.8 | 0.8 | 2.7 | 1.0 | 1.5 | 80.1 | < LOD | < LOD |
| P1 | 12.3 | 1.6 | 3.7 | 82.5 | 8.0 | 4.3 | < LOD | 2.7 | 1.0 | 1.9 | 80.6 | < LOD | < LOD |
| Q1 | 13.7 | 1.8 | 4.1 | 80.4 | 9.0 | 4.7 | < LOD | 3.0 | 1.1 | 1.2 | 79.2 | < LOD | < LOD |
| R1 | 12.9 | 1.8 | 4.4 | 80.9 | 8.2 | 4.6 | < LOD | 3.2 | 1.2 | 1.5 | 79.4 | < LOD | < LOD |
| S1 | 12.3 | 1.9 | 3.9 | 82.0 | 7.9 | 4.4 | < LOD | 2.6 | 1.3 | 1.4 | 80.5 | < LOD | < LOD |
| O2 | 13.2 | 2.2 | 4.7 | 74.4 | 8.1 | 5.1 | < LOD | 2.9 | 1.9 | 1.0 | 73.3 | 4.3 | 1.3 |
| P2 | 13.2 | 2.1 | 4.2 | 71.9 | 8.1 | 5.2 | < LOD | 2.4 | 1.7 | 0.9 | 71.0 | 7.2 | 1.4 |
| Q2 | 12.2 | 2.1 | 4.0 | 74.2 | 7.3 | 4.9 | < LOD | 2.4 | 1.7 | 0.7 | 73.6 | 6.4 | 1.1 |
| R2 | 12.9 | 2.6 | 3.9 | 66.1 | 6.9 | 5.4 | 0.6 | 2.5 | 1.4 | 0.8 | 65.3 | 9.8 | 4.8 |
| BMH-6 | 13.7 | 1.4 | 4.9 | 77.8 | 7.4 | 6.4 | < LOD | 2.8 | 2.1 | 0.8 | 77.0 | 2.2 | < LOD |
| BMH-7 | 13.7 | 1.9 | 5.2 | 78.4 | 8.0 | 4.6 | 1.1 | 3.6 | 1.6 | 1.0 | 77.4 | 0.8 | < LOD |
| BMH-8 | 13.3 | 1.7 | 4.3 | 79.7 | 8.0 | 5.3 | < LOD | 2.7 | 1.6 | 1.4 | 78.4 | 1.0 | < LOD |
| BMH-9 | 14.1 | 1.8 | 4.7 | 79.4 | 9.0 | 5.1 | < LOD | 3.2 | 1.5 | 1.6 | 77.8 | < LOD | < LOD |
| BMH-10 | 15.5 | 1.6 | 4.4 | 78.5 | 8.8 | 5.8 | 1.0 | 3.1 | 1.3 | 1.1 | 77.3 | < LOD | < LOD |
| BMH-11 | 14.1 | 2.0 | 4.7 | 79.2 | 9.1 | 5.0 | < LOD | 3.3 | 1.4 | 1.2 | 77.9 | < LOD | < LOD |
| BMH-12 | 11.6 | 1.6 | 4.8 | 78.8 | 6.3 | 5.3 | < LOD | 3.0 | 1.9 | 1.3 | 77.5 | 3.2 | < LOD |
| BMH-13 | 13.1 | 1.3 | 4.8 | 78.7 | 8.0 | 5.1 | < LOD | 2.7 | 2.1 | 1.2 | 77.5 | 2.1 | < LOD |
| BMH-14 | 13.8 | 1.1 | 5.1 | 78.5 | 7.3 | 5.7 | 0.8 | 3.3 | 1.8 | 1.3 | 77.2 | 1.6 | < LOD |
| BMH-15 | 14.3 | 1.5 | 5.2 | 78.1 | 7.4 | 5.5 | 1.5 | 3.1 | 2.1 | 1.4 | 76.7 | 0.9 | < LOD |
| BMH-16 | 12.5 | 1.1 | 4.9 | 81.6 | 8.1 | 4.4 | < LOD | 2.8 | 2.1 | 0.9 | 80.6 | < LOD | < LOD |
| BMH-17 | 13.2 | 1.5 | 4.8 | 79.5 | 7.3 | 5.9 | < LOD | 3.0 | 1.8 | 1.4 | 78.1 | 0.9 | < LOD |
| BMH-18 | 13.1 | 1.1 | 5.2 | 79.3 | 7.5 | 4.7 | 0.9 | 3.2 | 1.9 | 1.4 | 77.9 | 1.4 | < LOD |
| BMH-19 | 13.3 | 1.2 | 4.8 | 78.6 | 7.8 | 5.5 | < LOD | 3.0 | 1.7 | 1.5 | 77.1 | 2.2 | < LOD |

**Table S15 -** Linkage region and sulfation degree of 39 bovine mucosa heparin samples

| Sample | LR | oxSer | DSulf |
|---|---|---|---|
| BMH-1 | 3.0 | 100.0 | 2.26 |
| BMH-2 | 2.8 | 74.6 | 2.25 |
| BMH-3 | 1.8 | 100.0 | 2.27 |
| BMH-4 | 1.6 | 100.0 | 2.28 |
| BMH-5 | 1.8 | 100.0 | 2.28 |
| E1 | 1.7 | 100.0 | 2.29 |
| F1 | 3.3 | 100.0 | 2.26 |
| G1 | 2.4 | 79.5 | 2.28 |
| H1 | 2.8 | 100.0 | 2.30 |
| E2 | 2.4 | 100.0 | 2.30 |
| F2 | 4.1 | 100.0 | 2.16 |
| G2 | 5.7 | 30.2 | 2.15 |
| K1 | 4.1 | 38.9 | 2.13 |
| L1 | 2.1 | 100.0 | 2.25 |
| M1 | 1.0 | 100.0 | 2.29 |
| N1 | 1.0 | 100.0 | 2.31 |
| O1 | 1.3 | 100.0 | 2.33 |
| P1 | 1.1 | 100.0 | 2.33 |
| Q1 | 1.5 | 100.0 | 2.30 |
| R1 | 2.1 | 100.0 | 2.26 |
| S1 | 2.2 | 100.0 | 2.28 |
| O2 | 1.8 | 100.0 | 2.31 |
| P2 | 2.6 | 100.0 | 2.30 |
| Q2 | 4.4 | 54.8 | 2.25 |
| R2 | 3.3 | 100.0 | 2.28 |
| BMH-6 | 4.1 | 64.1 | 2.22 |
| BMH-7 | 4.0 | 42.0 | 2.21 |
| BMH-8 | 2.0 | 100.0 | 2.30 |
| BMH-9 | 1.6 | 100.0 | 2.28 |
| BMH-10 | 2.1 | 100.0 | 2.29 |
| BMH-11 | 1.7 | 100.0 | 2.27 |
| BMH-12 | 2.8 | 100.0 | 2.28 |
| BMH-13 | 2.2 | 100.0 | 2.29 |
| BMH-14 | 3.2 | 100.0 | 2.29 |
| BMH-15 | 3.7 | 100.0 | 2.34 |
| BMH-16 | 1.7 | 100.0 | 2.28 |
| BMH-17 | 3.7 | 100.0 | 2.30 |
| BMH-18 | 3.2 | 100.0 | 2.33 |
| BMH-19 | 2.8 | 63.2 | 2.29 |

**Table S16-** Molar percentual of glucosamine residues of 7 beef lung heparin samples

| Sample | ANH2,6x | ANAc,6x | ANS,3S,6x | ANS,6X | ANAc,6X-G | ANAc,6X-I | ANS,6X-G | ANS,6X-I | ANS,6X-I2S | ANS,6X-GalA | ANS,6X-Epox | ANAc,6XaRed | ANS,6XaRed | % 6S |
|---|---|---|---|---|---|---|---|---|---|---|---|---|---|---|
| BLH-1 | 0.5 | 1.7 | 2.9 | 94.8 | 1.7 | < LOD | 5.2 | 3.7 | 83.5 | 1.4 | < LOD | < LOD | 1.1 | 90.3 |
| BLH-2 | 0.5 | 1.9 | 2.6 | 94.9 | 1.9 | < LOD | 7.2 | 4.2 | 79.3 | 0.9 | < LOD | < LOD | 3.2 | 90.2 |
| BLH-3 | 0.7 | 2.9 | 2.9 | 93.4 | 2.9 | < LOD | 7.5 | 3.5 | 80.3 | < LOD | < LOD | < LOD | 2.2 | 89.4 |
| BLH-4 | 0.6 | 2.2 | 3.5 | 93.7 | 1.7 | 0.5 | 7.8 | 3.9 | 78.8 | 0.8 | < LOD | < LOD | 2.5 | 88.9 |
| BLH-5 | 0.6 | 3.1 | 2.4 | 93.9 | 3.1 | < LOD | 8.3 | 4.4 | 77.5 | 0.8 | < LOD | < LOD | 2.8 | 87.8 |
| BLH-6 | 0.8 | 3.5 | 3.3 | 92.4 | 3.5 | < LOD | 7.7 | 3.1 | 79.4 | < LOD | < LOD | < LOD | 2.2 | 88.6 |
| BLH-7 | 0.8 | 3.2 | 3.5 | 92.5 | 2.7 | 0.5 | 7.8 | 4.5 | 77.5 | 0.6 | < LOD | < LOD | 2.1 | 88.4 |

**Table S17 -** Molar percentual of uronic acid residues of 7 beef lung heparin samples

| Sample | G2OH | G2S | I2OH | I2S | G-ANAc,6X | G-ANS,6X | G-ANS,3S,6X | I-ANY | I-ANY,6S | I2S-ANH2,6X | I2S-ANY,3X,6X | GalA | Epox |
|---|---|---|---|---|---|---|---|---|---|---|---|---|---|
| BLH-1 | 2.7 | < LOD | 2.1 | 95.3 | 0.5 | 1.4 | 0.8 | < LOD | 2.1 | 0.8 | 94.4 | < LOD | < LOD |
| BLH-2 | 4.6 | 0.4 | 2.9 | 92.1 | 1.7 | 2.0 | 0.8 | 0.8 | 2.1 | 1.4 | 90.7 | < LOD | < LOD |
| BLH-3 | 4.4 | 0.5 | 2.7 | 92.4 | 1.5 | 2.2 | 0.7 | 0.6 | 2.1 | 1.1 | 91.3 | < LOD | < LOD |
| BLH-4 | 4.5 | 0.5 | 2.6 | 92.4 | 1.7 | 2.2 | 0.6 | < LOD | 2.6 | 1.1 | 91.2 | < LOD | < LOD |
| BLH-5 | 5.4 | 0.7 | 3.5 | 90.3 | 2.0 | 2.6 | 0.9 | 1.0 | 2.6 | 1.1 | 89.3 | < LOD | < LOD |
| BLH-6 | 4.3 | < LOD | 3.2 | 92.5 | 1.6 | 2.7 | < LOD | 0.8 | 2.4 | 1.2 | 91.3 | < LOD | < LOD |
| BLH-7 | 5.4 | 0.6 | 3.3 | 90.8 | 1.8 | 2.6 | 1.0 | 0.9 | 2.4 | 1.9 | 88.9 | < LOD | < LOD |

**Table S18 -** Linkage region and sulfation degree of 7 Beef lung heparin samples

| Sample | LR | %ox Ser | DSulf |
|---|---|---|---|
| BLH-1 | < LOD | NA | 2.86 |
| BLH-2 | 1.3 | 100.0 | 2.83 |
| BLH-3 | 1.3 | 100.0 | 2.82 |
| BLH-4 | 1.1 | 10.00 | 2.82 |
| BLH-5 | 1.0 | 100.0 | 2.78 |
| BLH-6 | 1.5 | 64.0 | 2.80 |
| BLH-7 | 1.5 | 72.1 | 2.79 |

**Table S19 -** Molar percentual of glucosamine residues of 6 ovine mucosa heparin samples

| Sample | ANH2,6x | ANAc,6x | ANS,3S,6x | ANS,6X | ANAc,6X-G | ANAc,6X-I | ANS,6X-G | ANS,6X-I | ANS,6X-I2S | ANS,6X-GalA | ANS,6X-Epox | ANAc,6XaRed | ANS,6XaRed | % 6S |
|---|---|---|---|---|---|---|---|---|---|---|---|---|---|---|
| OMH-1 | < LOD | 5.2 | 7.3 | 87.5 | 4.5 | < LOD | 12.9 | 7.8 | 58.4 | 6.5 | < LOD | 0.7 | 2.0 | 89.6 |
| OMH-2 | < LOD | 4.9 | 5.4 | 89.7 | 4.1 | < LOD | 8.7 | 5.5 | 72.9 | 0.7 | < LOD | 0.8 | 2.0 | 86.4 |
| OMH-3 | 0.9 | 5.1 | 5.7 | 88.3 | 4.2 | < LOD | 10.1 | 5.0 | 70.4 | 1.4 | < LOD | 0.9 | 1.4 | 86.7 |
| OMH-4 | < LOD | 4.5 | 5.5 | 90.0 | 3.7 | < LOD | 9.9 | 5.5 | 71.0 | 1.6 | < LOD | 0.8 | 2.0 | 87.9 |
| OMH-5 | 1.1 | 5.8 | 6.6 | 86.5 | 4.7 | < LOD | 11.0 | 6.1 | 68.1 | < LOD | < LOD | 1.1 | 1.4 | 86.9 |
| OMH-6 | 0.9 | 7.1 | 7.2 | 84.8 | 5.8 | < LOD | 11.2 | 5.6 | 65.5 | 0.7 | < LOD | 1.3 | 1.8 | 86.0 |

**Table S20 -** Molar percentual of uronic acid of 6 ovine mucosa heparin samples

| Sample | G2OH | G2S | I2OH | I2S | G-ANAc,6X | G-ANS,6X | G-ANS,3S,6X | I-ANY | I-ANY,6S | I2S-ANH2,6X | I2S-ANY,3X,6X | GalA | Epox |
|---|---|---|---|---|---|---|---|---|---|---|---|---|---|
| OMH-1 | 11.98 | < LOD | 6.83 | 71.41 | 2.31 | 6.84 | 2.83 | 0.7 | 6.13 | < LOD | 71.41 | 9.78 | < LOD |
| OMH-2 | 7.8 | < LOD | 5.1 | 87.1 | 1.4 | 5.1 | 1.3 | 0.6 | 4.6 | 0.8 | 86.4 | < LOD | < LOD |
| OMH-3 | 6.9 | < LOD | 5.2 | 87.9 | 1.6 | 4.4 | 0.9 | 0.9 | 4.3 | 1.2 | 86.7 | < LOD | < LOD |
| OMH-4 | 7.3 | < LOD | 6.1 | 86.6 | 1.5 | 4.8 | 1.0 | 0.9 | 5.2 | < LOD | 86.6 | < LOD | < LOD |
| OMH-5 | 7.8 | < LOD | 5.9 | 86.4 | 2.3 | 4.4 | 1.0 | 0.8 | 5.1 | 1.5 | 84.9 | < LOD | < LOD |
| OMH-6 | 8.2 | < LOD | 6.0 | 85.9 | 2.6 | 4.7 | 0.9 | 0.7 | 5.2 | 1.2 | 84.7 | < LOD | < LOD |

**Table S21 -** Linkage region and sulfation degree of 6 ovine mucosa heparin samples

| Sample | LR | %ox Ser | DSulf |
|---|---|---|---|
| OMH-1 | 1.4 | 100.0 | 2.63 |
| OMH-2 | 2.5 | 26.4 | 2.74 |
| OMH-3 | 2.7 | 0.0 | 2.74 |
| OMH-4 | 2.6 | 0.0 | 2.76 |
| OMH-5 | 3.8 | 27.9 | 2.73 |
| OMH-6 | 3.4 | 42.5 | 2.71 |
